# The Escitalopram versus Electric Current Therapy for Treating Depression Clinical Study (ELECT-TDCS): rationale and study design of a non-inferiority, triple-arm, placebo-controlled clinical trial: Estudo clínico Escitalopram *versus* Eletroterapia no Tratamento da Depressão (ELECT-TDCS): racional e desenho de estudo de um ensaio de não inferioridade, de três braços, placebo-controlado

**DOI:** 10.1590/1516-3180.2014.00351712

**Published:** 2014-12-19

**Authors:** André Russowsky Brunoni, Bernardo Sampaio-Junior, Adriano Henrique Moffa, Lucas Borrione, Barbara Schwair Nogueira, Luana Vanessa Marotti Aparício, Beatriz Veronezi, Marina Moreno, Raquel Albano Fernandes, Diego Tavares, Priscila Vilela Silveira Bueno, Ole Seibt, Marom Bikson, Renerio Fraguas, Isabela Martins Benseñor

**Affiliations:** I MD, PhD. Attending Physician, Interdisciplinary Neuromodulation Service, Interdisciplinary Center for Applied Neuromodulation, Hospital Universitário (HU), and Service of Interdisciplinary Neuromodulation, Laboratory of Neurosciences (LIM-27), Department and Institute of Psychiatry, Universidade de São Paulo (USP), São Paulo, Brazil.; II MD. Interdisciplinary Neuromodulation Service, Interdisciplinary Center for Applied Neuromodulation, HU, and Service of Interdisciplinary Neuromodulation, Laboratory of Neurosciences (LIM-27), Department and Institute of Psychiatry, USP, São Paulo, Brazil.; III BA, MSc. Interdisciplinary Center for Applied Neuromodulation, HU, USP, São Paulo, Brazil.; IV MD, MSc. Interdisciplinary Center for Applied Neuromodulation, HU and Department and Institute of Psychiatry, USP, São Paulo, Brazil.; V MD, MSc. Interdisciplinary Center for Applied Neuromodulation, HU, USP, São Paulo, Brazil.; VI Psychology Student, Interdisciplinary Center for Applied Neuromodulation, HU, USP, São Paulo, Brazil.; VII MD. Medical Resident and Student, Interdisciplinary Neuromodulation Service, Interdisciplinary Center for Applied Neuromodulation, HU and Department and Institute of Psychiatry, USP, São Paulo, Brazil.; VIII MSc. Researcher, Department of Biomedical Engineering, City College of City University of New York, New York, USA.; IX PhD. Head, Department of Biomedical Engineering, City College of City University of New York, New York, USA.; X MD, PhD. Assistant Professor, HU and Department and Institute of Psychiatry, USP, São Paulo, Brazil.; XI MD, PhD. Assistant Professor, HU, USP, São Paulo, Brazil.

**Keywords:** Depressive disorder, major, Electric stimulation therapy, Citalopram, Randomized controlled trial, Biological markers, Transtorno depressivo maior, Terapia por estimulação elétrica, Citalopram, Ensaio clínico controlado aleatório, Marcadores biológicos

## Abstract

**CONTEXT AND OBJECTIVE:**

Major depressive disorder (MDD) is a common psychiatric condition, mostly treated with antidepressant drugs, which are limited due to refractoriness and adverse effects. We describe the study rationale and design of ELECT-TDCS (Escitalopram versus Electric Current Therapy for Treating Depression Clinical Study), which is investigating a non-pharmacological treatment known as transcranial direct current stimulation (tDCS).

**DESIGN AND SETTING:**

Phase-III, randomized, non-inferiority, triple-arm, placebo-controlled study, ongoing in São Paulo, Brazil.

**METHODS:**

ELECT-TDCS compares the efficacy of active tDCS/placebo pill, sham tDCS/escitalopram 20 mg/day and sham tDCS/placebo pill, for ten weeks, randomizing 240 patients in a 3:3:2 ratio, respectively. Our primary aim is to show that tDCS is not inferior to escitalopram with a non-inferiority margin of at least 50% of the escitalopram effect, in relation to placebo. As secondary aims, we investigate several biomarkers such as genetic polymorphisms, neurotrophin serum markers, motor cortical excitability, heart rate variability and neuroimaging.

**RESULTS:**

Proving that tDCS is similarly effective to antidepressants would have a tremendous impact on clinical psychiatry, since tDCS is virtually devoid of adverse effects. Its ease of use, portability and low price are further compelling characteristics for its use in primary and secondary healthcare. Multimodal investigation of biomarkers will also contribute towards understanding the antidepressant mechanisms of action of tDCS.

**CONCLUSION:**

Our results have the potential to introduce a novel technique to the therapeutic arsenal of treatments for depression.

## RESUMO

**CONTEXTO E OBJETIVO:**

O transtorno depressivo maior (TDM) é uma condição psiquiátrica comum, tratada com medicamentos antidepressivos, os quais são limitados devido à refratariedade e efeitos adversos. Descrevemos o racional e o desenho do Estudo Clínico Escitalopram *versus* Eletroterapia no Tratamento da Depressão (ELECT-TDCS), que investiga um tratamento não farmacológico, conhecido como estimulação transcraniana por corrente contínua (ETCC).

**DESENHO E LOCAL:**

Ensaio de fase III, randomizado, de não inferioridade, de três braços, placebo-controlado, em execução em São Paulo, Brasil.

**MÉTODOS:**

O estudo compara a eficácia da ETCC ativa/pílula placebo, ETCC simulada/escitalopram 20 mg/dia e ETCC simulada/pílula placebo durante 10 semanas, randomizando 240 pacientes em uma proporção 3:3:2, respectivamente. O objetivo principal é demostrar que a ETCC não é inferior ao escitalopram com uma margem de não inferioridade de pelo menos 50% do efeito de escitalopram em relação ao placebo. Como objetivos secundários, investigamos biomarcadores como polimorfismos genéticos, marcadores séricos, excitabilidade cortical motora, variabilidade da frequência cardíaca e neuroimagem.

**RESULTADOS:**

Provar que ETCC é igualmente eficaz a antidepressivos teria um tremendo impacto na psiquiatria clínica, uma vez que a ETCC é praticamente isenta de efeitos adversos. Sua facilidade de uso, portabilidade e preço baixo são outras características atraentes para uso na atenção primária e secundária de saúde. A investigação multimodal de biomarcadores também contribuirá para a compreensão dos mecanismos de ação antidepressivos da ETCC.

**CONCLUSÃO:**

Os nossos resultados podem introduzir uma nova técnica no arsenal terapêutico do tratamento da depressão.

## INTRODUCTION

Major depressive disorder (MDD) is a psychiatric condition with high prevalence and recurrence worldwide,^1^ and it is projected to be the second greatest cause of disability worldwide in 2020.^2^ However, antidepressant drugs are only moderately effective for MDD treatment.^3^ Moreover, common adverse effects can lead to treatment discontinuation and recrudescence of symptoms.^4^ For these reasons, novel treatment strategies are continuously pursued.

In this context, non-invasive brain stimulation therapies (electroceuticals) have been increasingly investigated as non-pharmacological MDD treatments, such as repetitive transcranial magnetic stimulation (rTMS), a technique that discharges potent electromagnetic fields through a coil placed over the patient’s head.^5^ Repetitive TMS is an effective treatment for MDD,^6^^,^^7^ although issues such as high cost of application, discomfort and specialized technology limit widespread adoption.^8^

Transcranial direct current stimulation (tDCS) is a neuromodulatory technique that presents low cost, high portability and a benign profile of adverse effects and is relatively simple to use.^9^^,^^10^ It consists of applying a low-intensity electric current across the patient’s head using two electrodes on the scalp. In a seminal study in 2000, Nitsche and Paulus demonstrated that anodal and cathodal tDCS applied over the motor cortex respectively increased and decreased motor cortical excitability, as measured by means of TMS motor-evoked potentials,^11^ thereby showing that tDCS could alter cortical excitability.^12^ Its effects depend not only on the polarity of the current, but also on other factors, such as baseline cortical activity, neuronal orientation and delivered current intensity and duration.^13^^,^^14^^,^^15^

The use of tDCS for MDD is based on findings from neuroimaging studies (such as Mayberg et al.^16^), which suggest that the depressive state is associated with hypoactivity of prefrontal areas, particularly the left dorsolateral prefrontal cortex (DLPFC), and that it can be resolved through an increase in the activity of this area. Therefore, treatment strategies that increase left DLPFC activity might treat depressive symptoms. This approach has been adopted and validated in studies using high-frequency (excitatory) rTMS^6^ and anodal tDCS over the DLPFC.^17^ Alternatively, some trials have applied the cathode over the right DLPFC (such as Ferrucci et al.^18^), or have used low-frequency (inhibitory) rTMS over this region,^19^ in accordance with the prefrontal asymmetry theory of depression that states that the right DLPFC presents abnormally high activity during depressive episodes.^20^

Several non-controlled and controlled studies using tDCS for MDD have been published, from 2006 onwards.^17^ In the first randomized clinical trial (RCT) in this field, Fregni et al.^21^ showed the efficacy of active tDCS versus sham for ameliorating depressive symptoms, in a pilot study on 10 patients. More recent RCTs enrolling larger samples^22^^,^^23^ have shown that active tDCS was an effective treatment for MDD. Its efficacy was corroborated by a recent meta-analysis.^24^ However, exhaustive RCTs addressing tDCS efficacy are still needed, since some results from RCTs have been non-significant,^25^^,^^26^^,^^27^ the total number of subjects investigated is still low and the optimal parameter protocols for tDCS in MDD remain to be determined.

**Figure 1.**
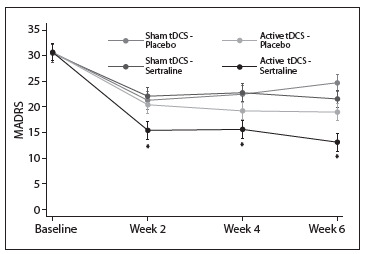
Main results of the SELECT-TDCS trial. The figure shows the primary outcome of the Sertraline versus Electric Current Therapy for Treating Depression Clinical Study (SELECT-TDCS).^23^ The x-axis represents depression scores measured using the Montgomery-Asberg depression rating scale (MADRS). The y-axis shows changes in depression scores over time according to the treatment group. Adapted from Brunoni et al.^23^

In a recent factorial, placebo-controlled trial named SELECT-TDCS (Sertraline versus Electric Current Therapy for Treating Depression Clinical Study), we assessed the efficacy of tDCS combined and compared with sertraline 50 mg/day for treating 120 depressed patients.^28^ After two weeks of treatment and at the endpoint, the combined treatment was statistically superior to the other groups in terms of depression improvement and response and remission rates. Transcranial DCS was also superior to the other groups at the endpoint and not statistically different from sertraline^23^ (Figure 1). The main findings from this study were the synergistic effects of the combination of tDCS and sertraline and the efficacy of tDCS as monotherapy. However, although sertraline and tDCS efficacy did not statistically differ, we could not extend these results to claim similar efficacy for tDCS and antidepressant pharmacotherapy, because of study limitations such as the low dose of sertraline used, the relatively short trial duration, the finding that the sertraline group was not superior to placebo and the relatively underpowered comparison of tDCS versus sertraline.

## OBJECTIVE

Therefore, whereas we showed in SELECT-TDCS that tDCS combined with pharmacotherapy could enhance improvement of depression, the main study aim of ELECT-TDCS (Escitalopram versus Electric Current Therapy for Treating Depression Clinical Study) is to compare the efficacy of tDCS with a fully dosed, first-line antidepressant treatment (escitalopram 20 mg/day).

In this paper, we describe the rationale, design and methodology of the ongoing ELECT-TDCS trial. The trial started in October 2013 and plans to enroll 240 patients by January 2017. As of November 2014, approximately 130 patients had already been recruited.

## METHODS

### Overview

The ELECT-TDCS trial randomizes patients into: sham-tDCS/placebo-pill (placebo group), sham-tDCS/escitalopram (escitalopram group) and active-tDCS/placebo-pill (tDCS group). It was approved by the Local and National Ethics Committee (CAAE:10173712.3.0000.0076) of the University Hospital and Clinics Hospital of the University of São Paulo and is registered in clinicaltrials.gov (NCT01894815). All study participants provide written, informed consent for participation in the study.

Our null hypothesis is that the improvement, i.e. the difference in efficacy between baseline and endpoint measurements of the tDCS group (µ_tDCS_) will be less than or equal to 50% (retention fraction *f* ≤ 50%) of the difference in the improvement between escitalopram and placebo (µ_drug_ - µ_placebo_). The study’s primary aim is to prove the alternative hypothesis, i.e. that this difference is greater than 50% (*f* > 50%).

$$H_{o}:\;{µ}_{tDCS}\;-\;{µ}_{placebo}\;\leq \;50\%\;({µ}_{drug}\;-\;{µ}_{placebo})$$

*or*

$$H_{o}:\;{µ}_{tDCS}\;-\;0.5\;{µ}_{placebo}\;-\;0.5\;{µ}_{drug}\;\leq \;0$$

$$H_{A}:\;{µ}_{tDCS}\;-\;{µ}_{placebo}>\;50\%\;({µ}_{drug}\;-\;{µ}_{placebo})$$

*or*

$$H_{A}:\;{µ}_{tDCS}\;-\;0.5\;{µ}_{placebo}\;-\;0.5\;{µ}_{drug}>\;0$$

In a non-inferiority triple-arm trial, there are three relevant comparisons: (1) experimental treatment versus active comparator (i.e. tDCS versus escitalopram); (2) experimental treatment versus placebo; and (3) active comparator versus placebo. Authors such as Koch and Röhmel^29^ have considered that the third comparison is not strictly necessary if the former two were significant, but in their approach, two or three null hypotheses would have to be independently rejected (thereby decreasing the P value), or the trial would only be valid if all hypotheses were rejected.^30^ Therefore, we use here an alternative model presented by Pigeot et al.^31^ and already used in the literature,^32^ in which the study aim can be presented in a single H_0_, which simultaneously tests the non-inferiority between active treatments and their superiority against placebo, thereby decreasing the number of multiple comparisons.

Our secondary aims are to explore the clinical improvement in terms of response status (more than 50% of improvement from baseline to endpoint) and remission status (HDRS-17 ≤ 7 at endpoint). We will also explore improvement in depression using the MADRS and the BDI. Finally, we will also investigate whether any early improvement (week 3) was observed between the groups.

We also aim to identify several predictors and mediators of tDCS response, as described below.

### Participants

We are recruiting patients of both genders, aged 18 to 75 years who have been diagnosed with major depressive disorder during an acute depressive episode, in accordance with the DSM-5 criteria (Diagnostic and Statistical Manual of Mental Disorders, 5^th^ edition).

The eligibility criteria include the presence of a depressive episode of at least moderate intensity (corresponding to a score ≥ 17 on the 17-item Hamilton Depression Rating Scale, HDRS-17), ability to read and understand Portuguese, at least eight years of schooling and availability to adhere to the study protocol. The exclusion criteria are: 1) other neuropsychiatric conditions, such as bipolar disorder, schizophrenia, substance dependence, dementia, traumatic brain injury, epilepsy and so forth (although participants with anxiety disorders can be included if the primary diagnosis is MDD); 2) high suicide risk (i.e. score > 2 in the Hamilton suicide question); 3) pregnancy; 4) specific contraindications against tDCS, such as electronic or metal implants in the cephalic segment; 5) specific contraindications against escitalopram; 6) severe/life-threatening clinical conditions; or 7) previous participation in other tDCS trials.

Participants will have to be either drug-naïve or drug-free regarding the use of antidepressant drugs (we considered an “antidepressant drug” to be any medication approved for treating MDD by the United States Food and Drug Administration as of the time of onset of the trial). A minimum period of 3 weeks (5 weeks for fluoxetine) will be set aside for drug washout. Benzodiazepine drugs will be allowed, although only at low doses (less than 20 mg/day of diazepam or equivalent). Also, since escitalopram is our active comparator, patients using (or who have used) escitalopram in the current depressive episode will not be included, since they would be escitalopram-resistant.

The adherence strategies for minimizing dropouts include: reimbursement of transportation costs; flexibility in the study schedule; allowance of up to four missed visits during the acute study phase and up to two missed visits during the weekly tDCS phase; reminders for patients regarding their appointments; and offering active tDCS after the endpoint to non-responder patients who received sham tDCS.

### Interventions

**Figure 2.**
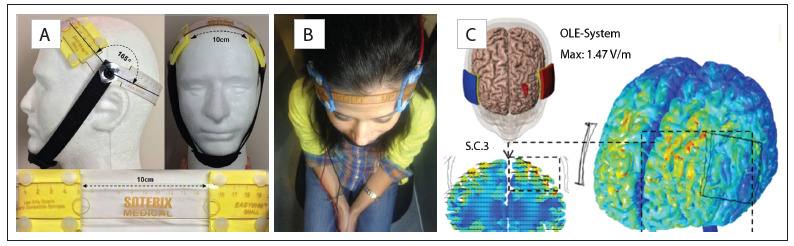
Electrode positioning in the ELECT-TDCS trial. (A) and (B) The figure shows the placement procedure for the Omni-Lateral-Electrode (OLE) system, which is placed as follows: 1) Select EasyStrap size (small, medium or large); 2) Place the midpoint of the occipital strap over inion (I_z_); 3) Position the hinges that link occipital-, electrode- and chin strap over the most dorsal point on the ear; 4) Adjust the angle between the occipital and electrode straps to 165° and the distance across the scalp between the dorsal electrode edges to 10 cm. Adapted from Seibt, Brunoni, Huang and Bikson (under review). (C) Brain current flow produced in one tDCS session in ELECT-TDCS.

We are using Soterix Medical tDCS devices specially customized for our study (Soterix Medical, New York, NY, USA, Model 1x1 tDCS-CT). For tDCS, the anode is placed over the left and the cathode over the right dorsolateral prefrontal cortex. The electrode positioning is based on the “Omni-Lateral-Electrode” (OLE) system, which is a simple, reproducible, and practical method for positioning the electrodes using a headband (Figures 2A and 2B). Moreover, the OLE method optimizes electric current densities across the DLPFC, in comparison with other non-neuronavigated methods for tDCS electrode placement, such as the EEG international 10-20 system and the 5-5 cm rule (Seibt, Brunoni, Huang and Bikson, under review), taking into consideration inter-subject variability in head anatomy (Figure 2C).

The stimulation parameters are: current intensity of 2 mA, electrode size of 25 cm^2^; session duration of 30 minutes (excluding the fade-in and fade-out periods of 15 seconds); and total number of 22 sessions, with 15 sessions applied consecutively once a day (except for weekends), and after that, seven more sessions applied once per week until the study endpoint at week 10. For operational reasons, these sessions are held on either Tuesdays or Thursdays, according to the patient’s preference and always respecting the one-week interval.

Sham tDCS is delivered using the same procedure as active tDCS, but using a period of only 30 seconds of active stimulation at 2 mA (or an overall active period of 60 seconds, taking into account the 15 seconds for both the fade-in and fade-out periods), with the stimulator remaining active but not generating current for 30 minutes, for the purpose of double blinding. This method, adapted from Gandiga et al.,^33^ has been used in several tDCS protocols^34^ and had the same blinding efficacy as the placebo pill in SELECT-TDCS.^35^ We are using fully automated devices that perform active or sham tDCS according to a randomized stimulation code.

The pharmacological intervention starts simultaneously with tDCS and consists of 10 mg pills of escitalopram oxalate or placebo. Patients receive 10 mg/day of escitalopram/placebo for the first 3 weeks and 20 mg/day for the remaining 7 weeks. Escitalopram was chosen because it is an effective antidepressant treatment with few adverse effects^36^ and its full dose (20 mg/day) is relatively easier to achieve than those of other antidepressant drugs. Thus, maximum dose-up titration can be done in the beginning of the trial. Therefore, we are able to compare tDCS against a full dose of an effective antidepressant without compromising blinding due to adverse effects.

The escitalopram pills are from Libbs (São Paulo, Brazil), a Brazilian pharmaceutical drug company that produces Reconter (escitalopram oxalate), which is a generic drug product comparable to the brand reference Lexapro (Lundbeck Brasil Ltda., Rio de Janeiro, Brazil). In Brazil, all generic drug products have their bioequivalence tested and certified by the Brazilian Health Surveillance Agency (ANVISA). We also independently assessed whether Reconter would be comparable to Lexapro in terms of dosage, strength and quality by testing both drugs in the Clinical Analysis Laboratory of the School of Pharmaceutical Sciences of the University of São Paulo. They achieved the same performance regarding their physical-chemical properties. The School of Pharmaceutical Sciences also produces the placebo pills, which are identical to the Reconter pills in terms of shape, color, weight and taste. Escitalopram and placebo pills are put in identical opaque bottles, identified solely by a number corresponding to the patient’s code. Adherence to the drug intervention will be verified by means of a pill count at the end of the study.

### Procedures

Participants are randomized in accordance with a computer-generated list at www.randomization.com. For allocation, we use opaque sealed envelopes containing the code corresponding to the group assigned for each participant. This code is entered into the tDCS device that automatically delivers either active or sham stimulation.

Diagnoses are made by certified psychiatrists or clinical psychologists and are confirmed through the Mini-International Neuropsychiatric Interview (M.I.N.I.). The data collected include diagnosis subtype (melancholic, atypical or depressive with mixed features), duration of illness and number of failed antidepressant treatments during the current episode. The Hamilton Depression Rating Scale (HDRS-17), the Montgomery-Asberg Depression Rating Scale (MADRS), the Positive and Negative Affect Scale (PANAS) and the STAI (State-Trait Anxiety Inventory) are applied at different time points (Table 1).

**Table 1.**
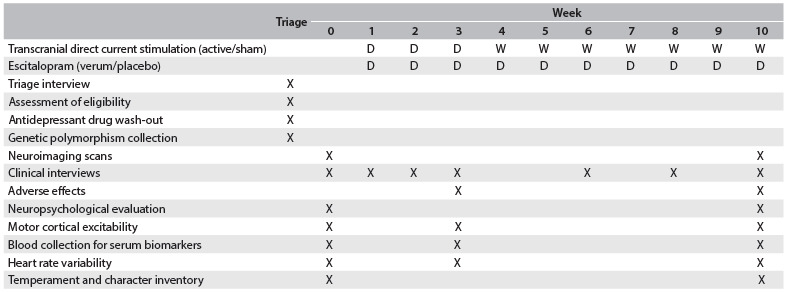
Study schedule for the ELECT-TDCS

“D” and “W” represent interventions performed daily and weekly, respectively.

To assess adverse effects, we use the Systematic Assessment for Treatment of Emergent Effects (SAFTEE)^37^ and the tDCS adverse events questionnaire.^10^ We apply the Young Mania Rating Scale (YMRS) to assess hypomanic symptoms, which have already been described after tDCS antidepressant treatment.^38^^,^^39^^,^^40^^,^^41^ Also, we include neuropsychological assessments in our study, primarily to verify that tDCS will not be associated with cognitive impairment, using the Montreal Cognitive Assessment (MOCA), the Wechsler Adult Intelligence Scale-III digit span (forward and backward) and digit symbol coding subtests, the Verbal Fluency Test (FAS and animal/fruit categories) and the Trail Making test. The Temperament and Character Inventory (TCI) is being collected at baseline and endpoint to assess the personality traits associated with and modified by the antidepressant treatment. We also assess blinding performance by asking patients to guess their own treatment allocation group at week 3 and at the endpoint, and ask interviewers to make the same guesses regarding these patients’ treatment.

Furthermore, we are investigating several biomarkers, including:

- Heart rate variability (HRV), which becomes lower in cases of depression^42^ and possibly reflects the disrupted sympathovagal balance observed in MDD. However, recent evidence suggests that this alteration is primarily driven by the direct effects of antidepressant drug treatment.^43^ In our SELECT-TDCS trial, we did not observe that either tDCS or sertraline changed HRV levels, which were lower than in matched healthy controls.^44^ In ELECT-TDCS, we assess HRV values over a larger timeframe (of 10 weeks) and in a larger sample size.
- Genetic polymorphisms: (i) The serotonin transporter gene (5HTTLPR, SLC6A4) is related to antidepressant response.^45^^,^^46^ Two out of three rTMS studies also found that it was associated with antidepressant response.^47^^,^^48^^,^^49^ In SELECT-TDCS we also found that it was associated with a larger active-sham difference.^50^ (ii) The 5HT_2A_ receptor gene (rs6311 and rs6313) was associated with a larger active-sham difference^51^ and antidepressant response.^52^ Although a meta-analysis was inconclusive regarding its association with antidepressant response,^46^ it was identified as associated with citalopram antidepressant response in the STAR*D trial.^53^ (iii) TPH1 polymorphism was associated with citalopram antidepressant response in the STAR*D trial. The A allele was associated with lower synthesis of serotonin^54^ and a recent meta-analysis^46^ showed that this allele was associated with worse antidepressant response. (iv) Brain-derived neurotrophic factor (BDNF) polymorphism (rs6265) is a neurotrophin associated with synaptic plasticity.^55^^,^^56^ Two meta-analyses showed that Met/Met and Val/Met were associated with antidepressant response,^46^^,^^57^ while one rTMS study found that Val/Val determined a better antidepressant response.^58^ In SELECT-TDCS, this polymorphism was not associated with antidepressant response.^50^
- Motor cortical excitability for tDCS has been used since the reappraisal of tDCS by Nitsche and Paulus in 2000.^11^ Currently, one important line of tDCS research involves measurement of motor cortex excitability after use of tDCS in combination with psychoactive drugs.^59^ This procedure allows indirect measurement of the GABAergic activity (ICI and CSP) and glutamatergic activity (ICF) of the motor system.^60^ These neurotransmitters are involved in the pathophysiology of depression.^61^ Recent studies observed that in relation to healthy subjects, the ICI, ICF and CSP indices are altered in depressed subjects,^62^^,^^63^^,^^64^ although their role as predictors of antidepressant response was not sufficiently investigated.
- Brain imaging by means of MRI will be collected in approximately half of the sample (120 patients) at baseline and endpoint, and this imaging will include voxel-based morphometry (VBM), diffusion tensor imaging (DTI) and resting-sate functional MRI. Regarding VBM, one consistent finding observed in depressed patients is that the volume of the grey matter in the prefrontal and anterior cingulate cortex bilaterally is lower,^65^^,^^66^^,^^67^ and that there is an increase in gray matter in the left DLPFC after successful antidepressant treatment.^68^ These brain areas will be explored as predictors of tDCS response. On DTI, the fractional anisotropy (FA) of the prefrontal cortex (bilaterally), right temporal lobe and right fusiform gyrus were found to be lower in MDD patients.^69^ Studies on repetitive TMS found an increase in FA in the left frontal-medial gyrus after successful antidepressant treatment,^70^^,^^71^ and these regions are explored in our study. For resting-state fMRI, we explore changes in the default-mode network (DMN) and anticorrelated network (AC) after tDCS treatment. Previous studies observed that depressed patients present greater DMN activity and lower AC activity^72^ and that antidepressant treatment can change the brain activity of these regions.^73^
- BDNF levels in blood are lower in depressed subjects than in healthy subjects and increase after successful pharmacological treatment.^74^^,^^75^ Nonetheless, recent meta-analyses showed that ECT increases BDNF levels in blood, but not rTMS or tDCS.^76^^,^^77^ The possible explanations for this are the low number of rTMS/tDCS studies that used non-optimal treatment protocols and the lower period of observation between measurements. In ELECT-TDCS, BDNF levels in blood are assessed at baseline, week 3 and the endpoint.

### Design and sample size determination

ELECT-TDCS uses a non-inferiority, triple-arm, placebo-controlled design, with 3:3:2 permuted block randomization in which participants are respectively assigned to escitalopram, tDCS or placebo. Our aim is to prove that tDCS is non-inferior (i.e. that it has similar or superior efficacy) to escitalopram. The placebo group is used for the following purposes. (1) To ensure assay sensitivity. The placebo ascertains that a similar result between escitalopram and tDCS occurred not due to a false-positive finding caused by methodological issues such as insufficient sample size, sample bias, poor blinding, etc., but in fact due to true equivalence between treatments. (2) To allow direct (superiority) comparisons between the pharmacological treatment and placebo, which is critical, since up to 50% of antidepressant drug trials fail to detect superiority between the active treatment and placebo.^78^ (3) To allow direct comparisons between the experimental treatment and placebo, given that evidence of tDCS efficacy is still being established. (4) To avoid setting up an arbitrary efficacy margin, which would be a problematic approach because the active comparator in the trial will not necessarily have the same efficacy as previous findings in the literature.^79^ For these reasons, the non-inferiority design with a placebo group is considered to be the gold standard for this type of trial according to the US Food and Drug Administration (FDA) and the European Medical Agency (EMA).^80^

The M_2_ margin (relative efficacy of the experimental treatment to the active comparator) is based on a fraction retention factor (*f)* of the M_1_, given that the relative efficacy of the experimental treatment to the active comparator could only be larger than the efficacy with placebo if the active comparator is worse than placebo. The fraction retention varies according to the condition investigated: for instance, in studies focusing on vaccines and oncology, the *f* value should be close to 1; whereas for chronic and functional disorders, this value ranges between one-half and one-third.^31^^,^^79^ There is no consensus regarding the *f* value in non-inferiority trials for MDD, although Nutt et al.^81^ proposed that for generalized anxiety disorder, the value should be 50%. In this trial, we adopted the *f* value of 50%, based on Nutt et al.^81^ and considering that MDD is comparable to generalized anxiety disorder with regard to methodological aspects (large placebo response, short duration of clinical trials, similar pharmacological treatments, comparable scales etc.).

Therefore, in our trial, M_2_/M_1_ = 0.5, although M_1_ is based on the difference between the active comparator and placebo and it will be known only when the study has been finished. Although this approach indexes the relative efficacy of tDCS versus escitalopram according to the placebo response of the study, it is theoretically possible that the escitalopram-placebo difference is too large, thus producing a large M_2_ value that could favor a finding that comparison between escitalopram and tDCS does not show any significant difference, even if escitalopram clinically outperforms tDCS. To avoid this issue, we will only consider that tDCS was non-inferior to escitalopram if the mean difference between escitalopram and tDCS is less than 3 points on the HDRS-17. The threshold of 3 points was chosen considering the National Institute for Health and Clinical Excellence (NICE) guidelines, which establish that this is a clinically meaningful difference in efficacy.^82^

To determine the sample size, we first needed to establish the randomization proportions between the groups. Pigeot et al.^31^ recommended a 1:1:k_p_ ratio, where k_p_ is determined according to the fraction retention factor, as shown below:

$$k_{placebo}\;=\;\left[(1\;-\;f)\;\surd 2\;+\;2f\right]/(1\;+\;f^{2})$$

According to this formula, for *f* = 0.5, k_p_ is 0.69. Therefore the optimal proportion for allocation between the groups is 1:1:0.69, or approximately 3:3:2, as used in ELECT-TDCS.

We estimated our sample size in accordance with the suggestion of Pigeot et al.^31^ for non-inferiority, triple-arm trials:

$$N_{arm}\geq {\left(t_{1-\alpha ,\;3n-3}\;+\;t_{1-\beta ,\;3n-3}\right)}^{2}*(1+f^{2}+{\left(1-f\right)}^{2})\;{*\left[\left(\sigma /\;{µ}_{DRUG}-{µ}_{PLACEBO})/(r-f\right)\right]}^{2*}$$

This formula shows that the sample size is a product of three factors (in parentheses). The factor in the first parentheses take into account the values of α and β, which were, respectively 0.025 (one-tailed) and 0.2. The factor in the second parentheses is the *f* value (the higher the *f* value is, the larger the necessary sample size will be), which was determined as 0.5 for our study. The factor in the last parentheses involves the standard deviation and effect sizes of the interventions. We took the value of the standard deviation to be a function of the difference in efficacy between the active comparator and the placebo:^31^

$$\sigma =\varepsilon \;({µ}_{DRUG}-{µ}_{PLACEBO})$$

The ε value indicates that the standard deviation is associated with the mean difference values observed in the study. It ranges from 0.2 to 2, and values > 1 are considered conservative. In the present study, we took the ε value to be 1.5, i.e. the standard deviation would be 1.5 times the value of the difference in efficacy between the active intervention and the placebo, which is compatible with depression studies in which the variance is usually high.^†^

Therefore, considering an attrition rate of 13% (similar to SELECT-TDCS), β = 0.2, α_one-tailed_ = 0.025, *f* = 0.5 and ε = 1.5, a total sample of 240 patients will be necessary in order to reject our null hypothesis. For 3:3:2 randomization, this means that in the end, 90, 90 and 60 patients will be respectively allocated to the tDCS, escitalopram and placebo groups.

### Statistical analysis

In accordance with the recommendations of Pigeot et al.^31^ and Rothmann et al.,^79^ we use a modified *t* test to address our primary study hypothesis (i.e. that tDCS is non-inferior to escitalopram). A *t* test involves obtaining a T value, which should be higher than (in a one-tailed test, as in our study) or different to (in a two-tailed test) a critical *t* value. The T value is obtained by dividing the difference in the means by the mean error (obtained according to the variance and sample size). For this analysis, the T value will be obtained as follows:

$$T=({µ}_{tDCS}-0.5{µ}_{PLACEBO}-0.5{µ}_{DRUG})/\sigma \surd (1/n_{TDCS}+0.25/n_{DRUG}+0.25/n_{PLACEBO})$$

or

$$T=7.44({µ}_{tDCS}-0.5{µ}_{PLACEBO}-0.5{µ}_{DRUG})/\sigma (considering\;that\;the\;n\;values\;are\;known)$$

This T value should be higher than the critical *t*, which for a one tailed α of 0.025 and 237 (N-3) degrees of freedom would be 1.97.

We will also perform exploratory analyses to identify whether a significant difference between the groups is observed over time. Therefore, we will perform mixed-model analyses of variance (ANOVAs) with one independent, within-subject variable (time, with three levels: baseline, week 3 and endpoint) and one independent, between-subject variable (group, with three levels). The dependent variables will be the scores from HDRS, MADRS or BDI. To determine whether a statistical significance is observed at a two-tailed p value of 0.05 or less, post-hoc analyses will be performed to analyze the main and interaction effects of our interventions. Exploratory analyses will also be performed to identify the influence of clinical, demographic and biological variables on the outcome. In addition, logistic regressions will be performed using response or remission as dependent variables and the group as an independent variable. Adverse effects will be assessed by counting the number of events in each group at each evaluation time. The chi-square test will be used to compare the frequency of adverse effects between the treatment groups.

For non-inferiority trials, it is unclear which approach (intention-to-treat, ITT; or per protocol, PP) should be used. ITT imputes data from study drop-outs and, although conservative for superiority trials, it can favor the hypothesis of lack of difference between groups in a non-inferiority design and, therefore, benefit (instead of penalizing) a non-inferiority design with methodological flaws. Nonetheless, neither the FDA^83^ nor the EMA^84^ clearly recommend the optimal approach and they suggest that both ITT and PP should be used. Mulla et al.^85^ also suggested that ITT should be used because it has the additional benefit of being more robust in relation to bias, compared with PP analysis. Therefore, missing data will be handled using an ITT approach and the findings will be confirmed through PP analysis. Finally, we will perform additional analyses according to the patients’ adherence to the study, using the categories of “fully adherent sample” (patients who missed or rescheduled two visits or fewer) and “completer sample” (patients who completed the study in accordance with the protocol). This approach was also used by George et al.^6^

## DISCUSSION

ELECT-TDCS will be the largest trial to date assessing the efficacy of tDCS and the first designed to specifically compare the efficacy of tDCS with a full dose of escitalopram. Demonstrating that tDCS presents efficacy similar to that of a pharmacological treatment is important, because antidepressant drugs present several relative and some absolute contraindications against use. Therefore, tDCS could increase the therapeutic arsenal for use among depressed patients who cannot or are not willing to use antidepressant drugs, e.g. pregnant women,^86^ HIV patients^87^ and patients presenting clinical conditions in which the pharmacokinetic interactions are problematic.^88^

Our study is also interesting from a cost-efficacy perspective, since tDCS is an affordable, portable and ease-to-use therapy. The cost of antidepressant therapy consisting of tDCS might, in fact, be comparable to that of antidepressant drugs, if it is taken into consideration that one tDCS device can be used to perform several applications per day and that one operator can deliver tDCS to two to three patients simultaneously. Finally, tDCS is a technique with few adverse effects.^10^ There are at present no reports of seizures or other severe life-threatening events. The most serious adverse effect reported hitherto is skin burn at the application site, and this has been an uncommon finding.^89^

Another key aspect of ELECT-TDCS is that several biomarkers will be assessed in a multimodal approach. tDCS does not seem to induce peripheral effects in cases of depression, such as changes to blood neurotrophic factors.^77^^,^^90^^,^^91^ However, it increases central neuroplasticity as indexed by paired associative stimulation,^92^ modulates cortical activity as indexed by electroencephalography^93^ and improves working memory and affective processing.^94^^,^^95^^,^^96^ Finally, since the same analyses will be performed in the escitalopram group, we will also explore whether the biological predictors and mediators are different between tDCS and escitalopram responders.

Our study protocol was designed by drawing on our experience from our earlier SELECT-TDCS trial and other advances observed in the field. The choice of using escitalopram at a dose of 20 mg/day represents an advance over the 50 mg/day dose of sertraline used in SELECT-TDCS. The decision to increase the total number of sessions from 12 to 22 was based on findings from a meta-analysis^24^ that suggested that a higher tDCS “dose” would be associated with a larger improvement in depression. Other changes in relation to SELECT-TDCS include: a longer wash-out period to ensure that the subjects were truly “antidepressant drug-free”; an increase in the maximum age of participants in order to improve recruitment; use of automated tDCS devices for sham stimulation, so as to improve blinding; a longer study duration to address the effects of tDCS over a longer time frame; and use of HDRS-17 (instead of MADRS) as the primary outcome measurement, given that in SELECT-TDCS, HDRS-17 presented lower variance and greater psychometric validity (Cronbach’s alpha and intraclass correlation) than MADRS, and was also more sensitive towards detecting between-group differences (data not published).

Nonetheless, most aspects of the SELECT-TDCS trial were maintained, such as recruitment of unipolar depressed patients with different degrees of refractoriness in an acute episode of at least moderate severity; enrollment of antidepressant-free patients; inclusion of patients using benzodiazepine drugs, since we considered that exclusion of these patients could harm recruitment and, in fact, be ineffective given that participants can retain information regarding use of these drugs; bilateral (left-right) DLPFC stimulation; and comparison with a placebo arm, which is crucial, given that as the efficacy of active versus sham tDCS is still under investigation.

### Limitations

ELECT-TDCS also presents some limitations. First, tDCS is being compared with escitalopram and therefore the results will not be fully generalizable to other antidepressant drugs. Second, we are not testing other tDCS montages and parameters that have also improved depressive symptoms. Third, we are not testing the combination of tDCS with pharmacotherapy or cognitive-behavioral therapy. Finally, our multimodal analyses are exploratory.

## CONCLUSIONS

The ELECT-TDCS trial is a 10-week, phase-III, non-inferiority, triple-arm, placebo-controlled study that is investigating whether tDCS is non-inferior (i.e. whether it has similar or superior efficacy) to 20 mg/day of escitalopram. The results from our trial have the potential to introduce a novel technique into the therapeutic arsenal for depression treatment, particularly in primary care contexts or among patients who cannot tolerate or are not willing to use antidepressant drugs. Our multimodal investigation of biomarkers will also contribute towards understanding the antidepressant mechanisms of the effects of tDCS.

## Footnotes

^†^
For comparative purposes, these values were as follows in SELECT-TDCS: µ_tDCS_ = 7.7, µ_PLACEBO_ = 3.6 and σ = 6.3 for the HDRS-17. Therefore, ε = 6.3/(7.7-3.6), or 1.53 (considering that µ_tDCS_ = µ_DRUG_).
